# Histopathological and molecular heterogeneity among individuals with dementia associated with Presenilin mutations

**DOI:** 10.1186/1750-1326-3-20

**Published:** 2008-11-20

**Authors:** Chera L Maarouf, Ian D Daugs, Salvatore Spina, Ruben Vidal, Tyler A Kokjohn, R Lyle Patton, Walter M Kalback, Dean C Luehrs, Douglas G Walker, Eduardo M Castaño, Thomas G Beach, Bernardino Ghetti, Alex E Roher

**Affiliations:** 1 The Longtine Center for Molecular Biology and Genetics, Sun Health Research Institute, Sun City, AZ 85351, USA; 2 Indiana Alzheimer Disease Center, Indiana University School of Medicine, Indianapolis, IN 46202, USA; 3 Department of Pathology and Laboratory Medicine, Indiana University School of Medicine, Indianapolis, IN 46202, USA; 4 Department of Neurological and Behavioral Sciences, University of Siena, Siena, Italy; 5 Department of Microbiology, Midwestern University, Glendale, AZ 85308, USA; 6 Laboratory of Neuroinflammation, Sun Health Research Institute, Sun City, AZ 85351, USA; 7 Fundacion Instituto Leloir, Buenos Aires, C1405BWE, Argentina; 8 WH Civin Laboratory of Neuropathology, Sun Health Research Institute, Sun City AZ 85351, USA

## Abstract

**Background:**

Mutations in the presenilin (*PSEN*) genes are associated with early-onset familial Alzheimer's disease (FAD). Biochemical characterizations and comparisons have revealed that many PSEN mutations alter γ-secretase activity to promote accumulation of toxic Aβ42 peptides. In this study, we compared the histopathologic and biochemical profiles of ten FAD cases expressing independent PSEN mutations and determined the degradation patterns of amyloid-β precursor protein (AβPP), Notch, N-cadherin and Erb-B4 by γ-secretase. In addition, the levels of Aβ40/42 peptides were quantified by ELISA.

**Results:**

We observed a wide variation in type, number and distribution of amyloid deposits and neurofibrillary tangles. Four of the ten cases examined exhibited a substantial enrichment in the relative proportions of Aβ40 over Aβ42. The AβPP N-terminal and C-terminal fragments and Tau species, assessed by Western blots and scanning densitometry, also demonstrated a wide variation. The Notch-1 intracellular domain was negligible by Western blotting in seven PSEN cases. There was significant N-cadherin and Erb-B4 peptide heterogeneity among the different PSEN mutations.

**Conclusion:**

These observations imply that missense mutations in *PSEN *genes can alter a range of key γ-secretase activities to produce an array of subtly different biochemical, neuropathological and clinical manifestations. Beyond the broad common features of dementia, plaques and tangles, the various PSEN mutations resulted in a wide heterogeneity and complexity and differed from sporadic AD.

## Background

Mutations in the presenilin-1 (*PSEN1*) and presenilin-2 (*PSEN2*) genes cause early-onset and aggressive forms of familial Alzheimer's disease (FAD). In humans the *PSEN1 *and *PSEN2 *genes are localized on chromosome 14 and chromosome 1, respectively, and encode for proteins with 65% amino acid sequence identity [1]. Presenilin-1 and PSEN2, 467 and 448 amino acids long, respectively, have nine transmembrane domains (TMD), two of which (TMD6 and TMD7) contain catalytic Asp residues at positions 257 and 385 forming an active center required for endoproteolysis [2,3]. More than 150 mutations in *PSEN1 *and *PSEN2 *have been reported ). The presenilins are part of γ-secretase, a heterotetrameric aspartyl membrane-bound protease complex comprised of four interacting molecules: PSEN, nicastrin, anterior pharynx defective 1 (Aph1) and presenilin enhancer 2 (Pen2) [4-6]. The biochemical and functional characterization of γ-secretase in recent years (reviewed in reference [7]) has permitted a better understanding of the hydrolysis of hydrophobic TMD and the important functional roles of their by-products. Gamma-secretase interacts with more than 25 different substrates, thus potentially participating in a wide range of cellular functions [8-11].

One of the numerous important substrates of the γ-secretase is the amyloid-beta precursor protein (AβPP), a type-1 membrane-bound molecule that is degraded by the action of the β- and γ-secretases to yield the 40/42 amino acid amyloid-β (Aβ) peptides. Gamma-secretase also hydrolyzes the AβPP at the ε-site to generate the transcription factor Aβ-intracellular domain (AICD) [12]. In AD, Aβ peptides deposit in the brain parenchyma and in the walls of the cerebral vasculature. The vast majority of FAD, resulting from mutations in the *AβPP *and *PSEN *genes, share the neuropathology observed in sporadic AD (SAD) which typically includes amyloid plaques and cerebral amyloid angiopathy, as well as neurofibrillary tangles (NFT) composed of hyperphosphorylated tau. It is widely accepted that mutations in the *PSEN *genes cause AD by influencing AβPP processing to yield Aβ42 preferentially [13]. Moreover the early age of clinical onset in FAD due to PSEN mutations appears to correlate with an increase in Aβ42 production and an associated decrease in Aβ40 genesis [14]. In addition, PSEN mutations appear to generate more Aβ42 than Aβ40 in transgenic mice and cultured cells [15-22]. This increase in the Aβ42/40 ratio due to PSEN mutations has been described as a 'gain of toxic function' [23]. However, in a recent publication, several of the PSEN mutations in transfected tissue culture cells secreted more Aβ40 than Aβ42 [24]. In addition, most PSEN mutations show reduced proteolytic activity on AβPP and a variety of other substrates, a phenotype that is recognized as a 'loss of function' [25]. Intriguingly, it has recently been established that total loss of PSEN1 and PSEN2 function in mice results in severe neurodegeneration analogous to that observed in AD, but without amyloid pathology [26].

Hyperphosphorylated forms of the microtubule associated protein tau are the major component of NFT, representing one of the pathologic hallmarks of SAD and FAD. Detailed chemical analyses of NFT has demonstrated substantial quantities of fatty acids, glycolipids and gangliosides, which suggest a membrane associated origin [27-30]. Electron microscopic studies have revealed that paired helical filaments (PHF) are intimately associated and probably derived from stacks of denatured cytomembranes such as smooth endoplasmic reticulum, Golgi and mitochondria [31,32].

One of the multiple proteins that γ-secretase hydrolyzes is Notch-1, a 2,532 amino acid transmembrane type-I protein (Swiss-Prot: P46531). In humans, the Notch family contains four highly conserved molecules (Notch1-4). Notch is a heterodimeric receptor involved in multiple and complex signaling pathways that include neuronal function and development, cardiovascular homeostasis, angiogenesis and cell regeneration [33-35]. Notch dysfunction elicits a large number of developmental defects and adult diseases such as different types of cancers [36]. Paralleling AβPP degradation, Notch-1 is cleaved at site S2 by the transmembrane ADAM/TACE proteases releasing the extracellular N-terminal region of the molecule that is bound to the Delta-type ligand on an opposing cell. Analogous to the AβPP γ/ε-sites, cleavage of Notch by the γ-secretase at sites S3 and S4, close to the cytoplasmic face of the membrane bi-layer, releases the Notch-1 Intracellular Domain (NICD). This 80 kDa C-terminal fragment is translocated into the nucleus, where it activates the ubiquitous transcription factor complex CBF1/Suppressor of Hairless/Lag1 (CSL), to increase the expression of a large number of genes [37-40].

Proteolytic degradation of N-cadherin, a molecule promoting neuronal cell adhesion is also mediated by γ-secretase. N-cadherin is an 881 amino acid type-I transmembrane calcium dependent molecule (Swiss-Prot: P19022) with five extracellular cadherin repeats and a short highly conserved cytoplasmic tail that interacts with the actin cytoskeleton through catenin molecules [41,42]. N-cadherin functions in cell-adhesion, embryogenesis, differentiation, migration, invasion, signal transduction, control of neuronal growth, synaptic configuration and neural plasticity [43-45]. Cleavage of N-cadherin by PSEN1 results in the generation of N-Cad/CTF2 which forms a complex with the transcriptional co-activator factor CBP (CREB binding protein) to repress transcription. It has been suggested that PSEN mutations lead to decreased N-Cad/CTF2 production, increased CBP/CREB mediated transcription activity and FAD [46].

Another important substrate of the presenilins is Erb-B4, a 1,283 amino acid type-I molecule (Swiss-Prot: Q15303), and a component of the epidermal growth factor (EGF) receptor that forms part of the family of tyrosine kinases [47]. Erb-B4 is involved in a wide range of cellular functions such as mitogenesis, differentiation, growth inhibition and survival [48]. Cleavage of Erb-B4 by tumor necrosis factor converting enzyme generates an ~80 kDa fragment that is subsequently hydrolyzed by γ-secretase to yield the cytoplasmic domain (B4ICD) that functions as a transcription factor [49-52]. In relation to AβPP metabolism, recent data suggests that the endogenous AICD is capable of binding to the promoter of the EGF receptor genes [53]. Important in this cascade of events are the neuroregulins (NRG), a group of neurotrophic factors that control tissue development, neuronal survival, synaptogenesis, microglial activation, astrocytic differentiation and oligodendrocyte survival by activating Erb-B receptors [54-56]. In SAD both NRG and Erb-B4 are associated with neuritic plaques [54].

In the present study, we compared neuropathological and biochemical findings among nine independent PSEN1 and one PSEN2 FAD cases, four SAD cases and two non-demented (ND) controls. A morphological account is given of the different types of Aβ plaques and NFT and their relative abundances. We determined Aβ40 and Aβ42 peptide levels, and the processing pattern and relative quantities of AβPP N-terminal and C-terminal peptides and soluble tau. We also investigated the differences among the PSEN mutations, as well as between the PSEN group and SAD or ND controls, with respect to Notch-1, N-cadherin and Erb-B4, molecules that are cleaved by the γ-secretase complex. These differences may reflect the effect of particular PSEN mutations on the multiple pathways in which the presenilins are involved. The significant differences among PSEN mutations are probably due to the position and the nature of PSEN amino acid substitutions, and the impact these mutations have upon multiple molecules involved in neuronal, glial and cerebrovascular homeostasis.

## Materials and methods

### Human tissues and neuropathology

Brains were removed from ten individuals carrying the following PSEN mutations; (PSEN1: A79V, A260V, F105L, Y115C, A431E, V261F, V261I, M146L and P264L, and PSEN2: N141I) and from four cases with SAD and two ND individuals. The postmortem interval (PMI) averages for the PSEN1/2 mutations, SAD and ND control subjects were 12.3 h, 10.8 h and 3.5 h, respectively. The discrepancy in PMI between the ND controls and SAD/PSEN patients is due to the different locations of tissue procurement. The ND controls were obtained from the Brain Bank at Sun Health Research Institute whose average PMI of 2.75 h is due partially to a rotating team that is on call 24 hours a day and the deaths occurring in the same community as the Institute [57]. Other centers, however, cannot shorten these times for multiple reasons. In particular, the PSEN brains were obtained from different laboratories that have different internal routines and time standards and had to be shipped on dry ice to the Indiana Alzheimer Disease Center. The average ages at death in the three cohorts were 53 years for the PSEN, 64 years for the SAD and 80 years for the ND group. The mean age at disease onset for the PSEN mutation subjects was 45 years and 52 years for the SAD cases. An attempt was made to use younger SAD individuals to make comparisons with the PSEN subjects more relevant.

In the PSEN group, five individuals were female and five were male. Three subjects were male and one individual was female in the SAD cohort. In the case of the ND controls, there was one male and one female. The left hemisphere from each case was fixed in 10% formalin, while the right hemisphere was frozen for genetic and biochemical studies. Fixed cerebral hemispheres were cut into coronal sections. For histological analyses, a sample of superior frontal and cingulate gyri were obtained for each case from sections at the level of the nucleus accumbens, corresponding to the section of frozen tissue from the contralateral hemisphere used for biochemical analysis. The tissue was dehydrated in a graded alcohol series, cleared in xylene and embedded in paraffin. Sections (8 μm) were processed according to the following methods: Weigert, hematoxylin-eosin, Woelcke-Heidenhain, Bodian and thioflavin-S. For immunohistochemical studies a panel of different antibodies was used. These included monoclonal antibody 3D6 against Aβ1-5 (Elan Corp., San Francisco, CA), monoclonal 10D5 against Aβ3-6 (Elan Corp.), monoclonal antibody 6E10 against Aβ1-14, monoclonal antibody 4G8 against Aβ16-24 (Signet Laboratories, Dedham, MA) and monoclonal antibody HYB310-01 against Aβ10-16 (Antibody Shop, Copenhagen, Denmark). Amyloid-β immunoreactive deposits in the frontal cortex, were classified into distinct morphological types as cotton-wool plaques (CWP), mature plaques, primitive plaques and diffuse plaques, as previously described [58,59]. Amyloid plaques and NFT were counted over three randomly selected non-overlapping 1 mm^2 ^microscopic fields.

### Aβ ELISAs

Brain tissue gray matter was homogenized in a 10× volume of 90% glass distilled formic acid (GDFA) with an electric grinder. The homogenates were centrifuged at 500,000 × *g *for 20 min at 4°C in a Beckman Optima TLA-ultracentrifuge using a 120.2 rotor (Beckman, Fullerton, CA). The supernatants were collected and dialyzed against a solution of 5 M guanidine HCl, 50 mM Tris HCl, pH 8.0. The samples were analyzed by ELISA using the anti-Aβ40 and anti-Aβ42 kits from Immunobiological Laboratories (Minneapolis, MN) and from Innogenetics (Gent, Belgium), respectively, and carried out following the manufacturer's instructions.

### Western Blot Analysis

Brain tissue was homogenized in a 10× volume of RIPA buffer (Sigma, St. Louis, MO; 150 mM NaCl, 1.0% IGEPAL^® ^CA-630, 0.5% sodium deoxycolate, 0.1% SDS 50 mM Tris, pH 8.0), containing a protease inhibitor cocktail (Roche Diagnostics, Mannheim, Germany) at 4°C. The homogenates were centrifuged at 14,000 × *g *for 20 min at 4°C in a Beckman Microfuge 22R centrifuge. The supernatants were collected and total protein quantified with a BCA protein assay kit (Pierce, Rockford, IL). The samples were placed in NuPage 2× LDS sample buffer (Invitrogen, Carlsbad, CA) that contained 50 mM dithiothreitol and heated at 80°C for 10 min. The proteins were electrophoresed on 4–12% Bis-Tris gels with NuPage 2-morpholinoethanesulfonic acid (MES) as the running buffer (Invitrogen). The proteins were then transferred onto 0.45 μm pore nitrocellulose membranes (Invitrogen) with NuPage transfer buffer (Invitrogen) and 20% methanol. The membranes were blocked with 5% non-fat milk in phosphate-buffered saline (PBS), 0.5% Tween 20 (Fluka, St. Louis, MO). Table 1 describes the primary and secondary antibodies used in the experiments. The proteins were visualized with SuperSignal WestPico Chemiluminescent (Pierce) substrate, CL-Xpose film (Pierce) and Kodak GBX developer (Sigma). The films were scanned and analyzed with a GS-800 calibrated densitometer (Bio-Rad, Hercules, CA) and Quantity One software (Bio-Rad). Antibodies were removed from the membranes with Restore™ Western Blot Stripping Buffer (Pierce). The blots were washed in PBS then blocked with 5% non-fat milk in PBS, 0.5% Tween 20 (Fluka, St. Louis, MO). Anti-mouse or anti-rabbit actin antibody was used to re-probe the membranes as protein normalization standards following the techniques described above. All values in the histograms have been corrected against actin abundance in all WB densities shown in this study.

**Table 1:** Primary and Secondary Antibodies Used in Western Blots

**Primary Antibody**	**Antigen Specificity**	**Secondary Antibody**	**Company/Catalog #**
22C11	AβPP aa. 66–81	M	Millipore/MAB348
CT9APP	Last 9 aa. of AβPP	R	Millipore/AB5352
Notch-1	NICD N-terminal 14 aa.	R	Millipore/AB5709
Erb-B4	C-terminal aa. 1258–1308	R	Santa Cruz/sc-283
N-Cadherin	aa. 802–819	M	BD Transduction Laboratories/610920
Tau	aa. 159–163	M	Pierce/MN1000
Actin Ab-5	Clone C4	M	BD Transduction Laboratories/A65020
Actin	N-terminus of human γ-actin	R	Abcam/Ab37063

AβPP = amyloid-β precursor protein; aa. = amino acid sequence; NICD = Notch-1 intracellular domain; M = goat-anti mouse IgG, HRP conjugated (Pierce); R = goat-anti rabbit IgG, HRP conjugated (Pierce)

### Apolipoprotein E Genotyping

DNA for apolipoprotein E (*APOE*) genotyping was extracted from pieces of cerebral cortex [60]. Tissue (50 mg) was digested to completion with proteinase K (1 mg/ml) at 55°C and purified using Wizard Genomic DNA purification kit (Promega, Madison WI). For each PCR reaction, approximately 500 ng of DNA from each sample was used. PCR primers and amplification conditions employed, and identification of *APOE *genotypes by HhaI restriction enzyme digestion of amplified material, were carried out according to published protocol [61]. To identify *APOEε *alleles, digested fragments were separated by electrophoresis through 9% acrylamide gels and revealed on ethidium bromide stained gels.

## Results

### I. Neuropathological findings and characterization of soluble Aβ and tau

A description of the most relevant demographic data and neuropathological features of the cases examined in the present study is shown in Table 2. In the PSEN mutation and SAD cases, neuronal loss and gliosis range from mild to severe. The two ND control individuals (81 year old female and 79 year old man), had no apparent gross brain atrophy and a sparse number of amyloid plaques with a low number of NFT and Braak scores of I (range I-VI). The individuals with PSEN mutations and SAD had an average brain weight of 1,042 g and 1,143 g, respectively, while the mean weight of the ND brains was 1180 g. In the PSEN mutation group, the *APOE *allele frequencies were ε2 = 0.10, ε3 = 0.80 and ε4= 0.10, while in the SAD group they were ε2 = 0, ε3 = 0.62 and ε4 = 0.38 (Table 2). The two ND individuals had ε2/ε3 and ε3/ε3 *APOE *genotypes.

**Table 2:** Patient Information and Neuropathology Data

**Case**	**Gender**	**Age at onset (yrs)**	**Age at death (yrs)**	***APOE *Genotype**	**Brain Weight (g)**	**Frontal Atrophy**	**Neuronal Loss**	**Gliosis**	**CAA**	*Avg CWP	*Avg Mature Plaques	*Avg Primitive Plaques	*Avg Diffuse Plaques	*Avg NFT
A79V-PSEN1	F	50	61	3/4	1060	2	2	2	0	0.00	8.67	43.67	65.33	9.00
A260V-PSEN1	M	40	46	2/3	1140	3	1/2	1/2	1	0.00	2.33	0.67	109.00	31.00
F105L-PSEN1	F	60	68	2/3	1070	2	2	2	1	0.00	1.00	0.00	78.67	17.00
Y115C-PSEN1	M	41	51	3/3	1026	2/3	2/3	2/3	1	0.00	0.67	8.67	70.67	31.33
A431E-PSEN1	F	35	43	3/3	764 PF	3	2/3	2/3	2	46.33	11.67	5.00	42.00	55.33
V261F-PSEN1	M	36	47	3/3	950	2	2	1	3	36.33	0.67	0.00	2.00	49.00
N141I-PSEN2	M	42	57	3/4	1136	2	1/2	1/2	2	0.00	2.00	1.33	67.67	65.00
V261I-PSEN1	F	48	55	3/3	1185	1	2	2	2	49.00	1.00	0.00	1.67	0.00
M146L-PSEN1	M	47	52	3/3	1150	1	2	2	1	0.00	14.00	0.00	102.00	14.33
P264L-PSEN1	F	48	53	3/3	940	2	2	2	0	13.67	8.00	0.00	12.67	14.67
sporadic AD	M	47	62	3/3	910	2	2/3	2/3	1	0.00	23.33	0.00	12.00	42.33
sporadic AD	M	55	66	3/4	1325	1	2/3	2/3	1	0.00	11.67	0.00	30.67	6.33
sporadic AD	M	58	73	3/4	1236 PF	2	2	2	2	0.00	5.67	0.00	15.33	12.00
sporadic AD	F	48	55	3/4	1100	3	2/3	2/3	0	0.00	36.67	0.00	21.33	55.33

**Figure 1:**
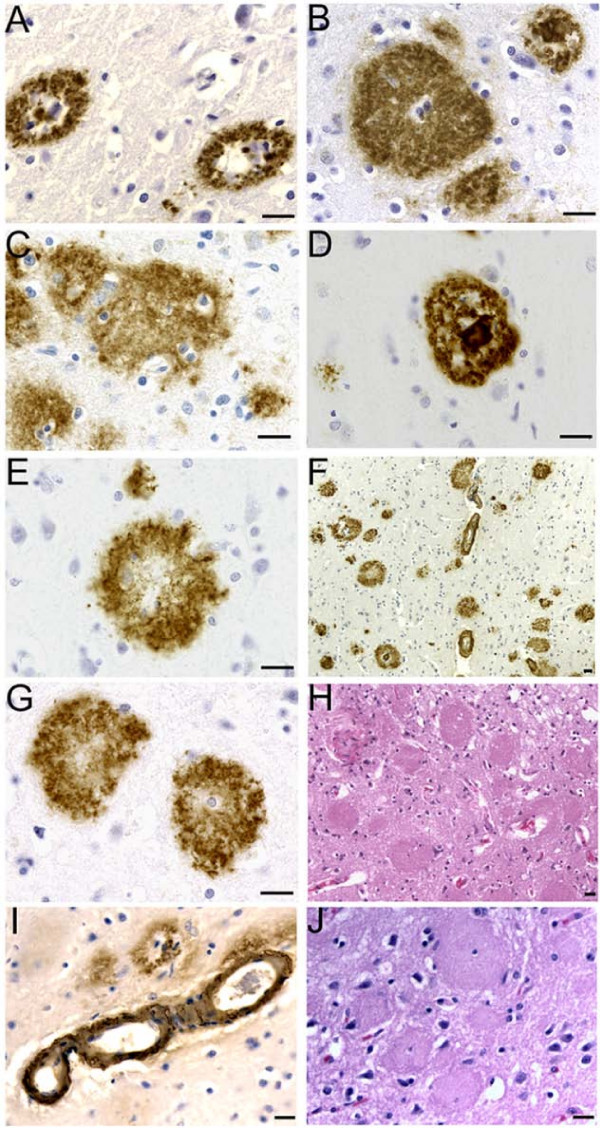
**Histology and immunocytochemistry of amyloid deposits in PSEN mutations**. A) PSEN2 N141I mutation primitive plaques immunoreacted with 10D5 antibody. B) Cotton wool plaques in the PSEN1 A431E mutation immunoreacted with the 21F12. On the upper-right corner there is a mature plaque. C) Cotton wool plaques associated with the PSEN1 A260V mutation immunoreacted with the 10D5 antibody. D) Mature neuritic plaque observed in the PSEN1 A79V mutation. 10D5 antibody. E) Primitive plaque localized the cerebral cortex of the PSEN1 A79V mutation immunoreacted with the 10D5 antibody. F) Cortical amyloid plaques immunoreacted with the 10D5 antibodies at low magnification in the PSEN1 V261F mutation. Numerous CWP and occasional mature core neuritic plaques are observed in addition to severe amyloid angiopathy. G) Cotton wool plaques in the PSEN1 V261F mutation developed with the 10D5 antibody. H) Abundant CWP observed in the PSEN1 V261F stained by hematoxilin and eosin. The plaques are surrounded by a discreet number of reactive astrocytes. In the remaining areas of the field several regions of severe neuronal loss and glyosis and moderate microvacuolization are observed. I) Severe Aβ immunoreactive cerebral amyloid angiopathy in the cerebral cortex of PSEN1 A431E mutation. J) A hematoxylin and eosin stained section of the cerebral cortex of PS1 431E mutation. Magnifications: 63 × (A, B, C, D, E, G); 40 × (I and J); 20 × (H) and 10 × (F). Scale Bars = 20 μm.

*all counts are measured in mm^2^; yrs = years; APOE = apolipoprotein E; g = grams; CAA = cerebral amyloid angiopathy; avg = average; CWP = cotton wool plaques; NFT = neurofibrillary tangles; F = female; M = males; yrs = years; AD = Alzheimer's disease; PSEN = presenilin; PF = post-fixed; 0 = none; 1 = mild; 2 = moderate; 3 = severe;

A quantitative comparison of the different type and number of neuropathological findings in the cerebral cortex of the PSEN mutation cases and the SAD cases is given in Table 2. A representative display of their morphology is shown in Figure 1. Cotton-wool plaques (CWP) were the most abundant type of Aβ deposit in four cases of *PSEN1 *mutations: A431E, V261F, V261I and P264L. They were observed throughout the cortical layers, although they were more abundant in the upper layers. The diameter of these plaques varied from an average of 80–90 μm in A431E, V261F and P264L cases to 145 μm in the V261I case. Their mean frequency was approximately 40–50 plaques/mm^2 ^in the A431E, V261F and V261I mutations. The P264L mutation case displayed a reduced number of CWP (average of 14/mm^2^), consistent with its overall reduced burden of Aβ deposition compared to that of the remaining cases. No CWP were observed in the superior frontal and cingulate gyri in the SAD group and in the cases with the PSEN1 mutations: A79V, A260V, F105L, Y115C and M146L and the PSEN2 N141I mutation.

Diffuse plaques, mostly prevalent in the intermediate cortical layers, constituted a common type of Aβ deposit. Their abundance varied substantially among the different mutations. They were most numerous in the A260V mutation (mean 109 plaques/mm^2^) and M146L mutation (102 plaques/mm^2^), but sparse in cases with V261F or V261I mutation (~2 plaques/mm^2^). Diffuse plaques were the predominant type of Aβ deposit in the PSEN1 mutations A79V, A260V, F105L, Y115C and M146L and the PSEN2 N141I mutation. The P264L mutation case displayed a similar number of CWP and diffuse plaques. The SAD group had an average of 19.8 plaques/mm^2 ^diffuse plaques (range 12.00–30.67).

Mature cored neuritic plaques were observed throughout all cortical layers, but were more frequent in the lower cortical layers. Their frequency varied among the different mutations, being higher in A431E and M146L PSEN1 mutations, and lower in the remaining cases. Neuritic plaques were less prevalent than CWP and diffuse plaques, and correlated approximately with the overall burden of Aβ deposition. They were only rarely observed in the cases with V261F or V261I mutation. In general, primitive plaques were abundant only in the PSEN1 A79V mutation with a few also seen in the upper cortical layers of Y115C and A431E PSEN1 mutations. Primitive plaques were not seen in the SAD cohort, while the mature plaques averaged 19.3 plaques/mm^2 ^(range 5.67–36.67/mm^2^).

The number of NFT grossly correlated with the overall burden of Aβ deposition. Nine of the PSEN cases displayed moderate to severe degrees of neurofibrillary degeneration (mean NFT value = 31.8/mm^2^; range 14.3 to 65/mm^2^), with the exception of the V261I mutation, in which sparse NFT were observed. Similar values were observed in the SAD cases (mean NFT value = 29.0/mm^2^; range 6.3 to 55.3/mm^2^). A negative slope correlation was observed between the number of NFT and disease duration (R = 0.70).

**Figure 2:**
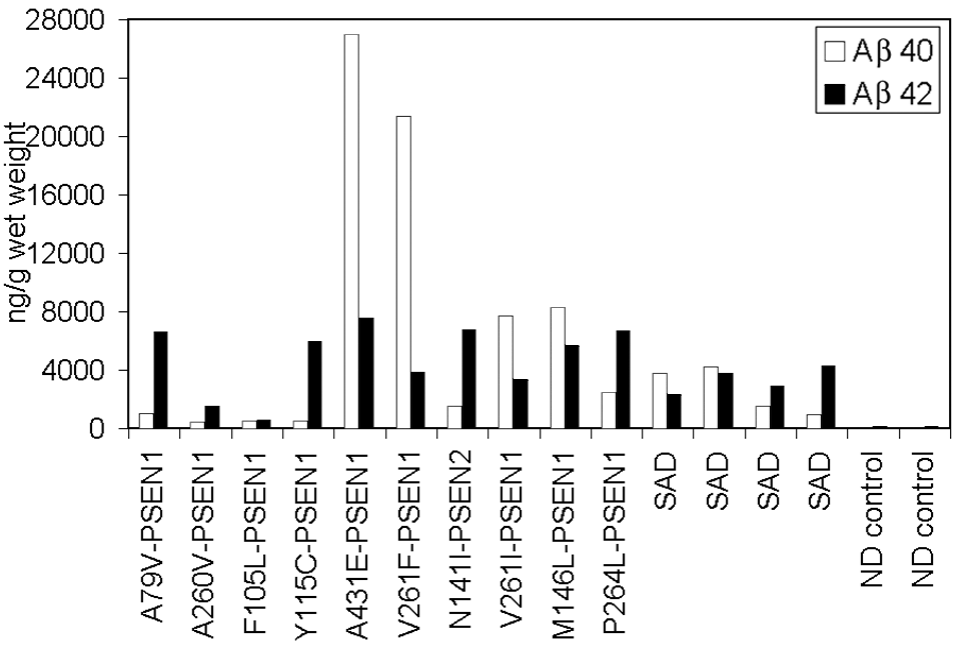
**Histogram depicting the relative amounts of Aβ40 and Aβ42 detected by ELISA**. Notice the abundant amount of Aβ40 in the PSEN A431E, V261F, V261I and M146L. The amounts of Aβ40 and Aβ42 were almost equivalent in case F105L. PSEN = presenilin; SAD = Alzheimer's disease; ND = non-demented.

**Figure 3:**
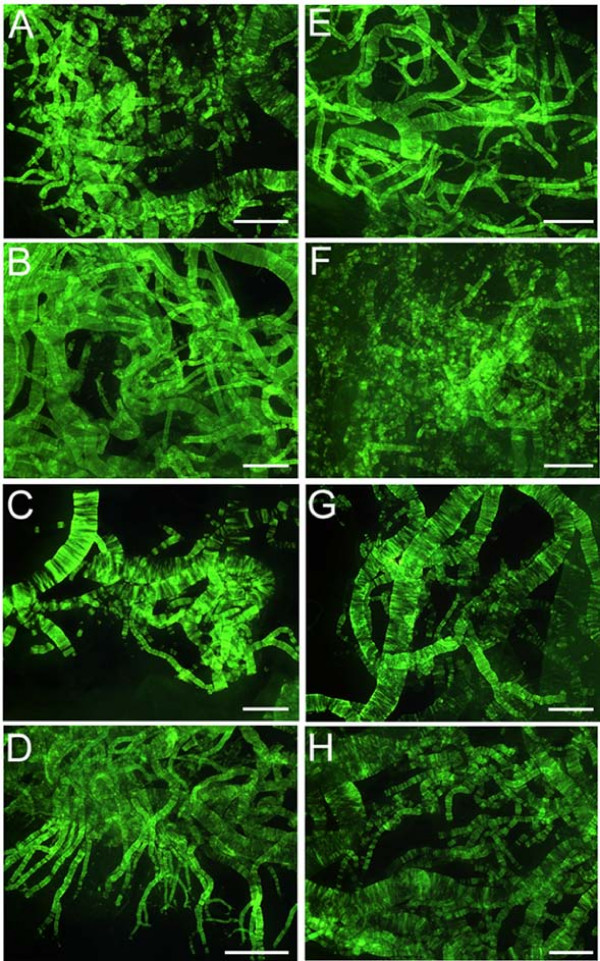
**Thioflavin-S histochemistry of leptomeningeal vessels**. Whole mounts of leptomeningeal vessels stained by thioflavine-S demonstrating amyloid angiopathy among the different PSEN mutations. A) PSEN1 M146L: moderate; B) PSEN1 V261I: severe; C) PSEN2 N141I: severe. D) PSEN1 V261F: severe; E) PSEN1 A431E: severe; F) PSEN1 Y115C: moderate; G) PSEN1 F105L: severe and H) PSEN1 A260V: moderate-severe. The remaining two PSEN1 mutations A79V and P264L had a mild amyloid deposition (data not shown). Scale Bars = 500 μm.

Quantification of Aβ levels in the cerebral cortex revealed that in four out of ten PSEN mutations Aβ40 was present in higher amounts than Aβ42 (Table 3 and Figure 2). Presenilin-1 cases with the A431E, V261F, V261I and M146L mutations had Aβ42/Aβ40 ratios of less than 1.00, being more prominent in the former two than in the latter two cases (0.28 and 0.18 versus 0.43 and 0.68). In one case (F105L), the ratio was close to 1:1 (Table 3). A similar pattern was found in two out of four SAD cases (Aβ42/Aβ40 ratios of 0.61 and 0.90). Cortical vascular amyloid in the four PSEN mutation cases cited above had scores of 2, 3, 2 and 1 (Table 2), where the scores 1, 2 and 3 correspond to mild, moderate and severe vascular amyloid deposits, respectively. In addition, three out of these four PSEN cases also had the highest values of CWP with the remaining individual (M146L) having a high number of diffuse deposits (102 per mm^2^) and mature plaques (14.00 per mm^2^) (Table 2). With the exception of PSEN cases A79V and P264L, in which very mild cortical vascular amyloid was evident in histological sections, all PSEN cases had mild to moderate amyloid angiopathy in the gray matter. Severe cortical amyloid angiopathy was only seen in the case of V261F. However, examination of isolated leptomeningeal vessels from all PSEN mutation cases demonstrated extensive vascular amyloidosis, as shown in Figure 3, which ranged from moderate (two cases) to moderate/severe (one case) to severe (five cases). In agreement with the cortical vascular amyloid assessment, cases A79V and P264L had negligible amounts of leptomeningeal vascular amyloid (data not shown). The amount of cortical vascular amyloid in the SAD cases ranged from none to moderate. The two ND cases had a mild form of CAA. In addition, we found that within the PSEN cohort positive correlation existed between levels of brain Aβ40 and degree of vascular amyloidosis (R = 0.81).

**Table 3:** Aβ ELISA Values

**Case**	**Aβ40 ng/g wet weight**	**Aβ42 ng/g wet weight**	**total Aβ ng/g wet weight**	**ratio Aβ42/Aβ40**
A79V-PSEN1	1039.59	6653.02	7692.60	6.40
A260V-PSEN1	442.61	1550.19	1992.81	3.50
F105L-PSEN1	497.31	575.18	1072.49	1.16
Y115C-PSEN1	531.35	5936.06	6467.41	11.17
A431E-PSEN1	27015.06	7530.96	34546.02	0.28
V261F-PSEN1	21373.91	3858.75	25232.66	0.18
N141I-PSEN2	1530.44	6734.30	8264.74	4.40
V261I-PSEN1	7736.66	3326.98	11063.64	0.43
M146L-PSEN1	8312.70	5680.64	13993.34	0.68
P264L-PSEN1	2452.49	6716.37	9168.86	2.74
Average	7093.21	4856.25	11949.46	3.09
SD	9560.57	2399.22	10429.32	3.52

sporadic AD	3815.67	2310.17	6125.84	0.61
sporadic AD	4235.19	3811.51	8046.71	0.90
sporadic AD	1524.04	2884.46	4408.50	1.89
sporadic AD	931.20	4280.58	5211.78	4.60
Average	2626.53	3321.68	5948.21	2.00
SD	1642.32	889.52	1565.06	1.82

ND control	40.73	148.30	189.03	3.64
ND control	42.52	114.79	157.31	2.70
Average	41.62	131.55	173.17	3.17
SD	1.27	23.69	22.43	0.67

PSEN = presenilin; SD = standard deviation; AD = Alzheimer's disease; ND = non-demented

Overall, the neuropathological distribution of the different types of plaques, the intensity of cerebrovascular amyloid and the number of NFT substantially differed among the nine PSEN1 and one PSEN2 mutations as well as from the four SAD cases used as pathology comparison controls. A detailed account of these parameters is given in Table 2 and Figures 1 and 3.

**Figure 4:**
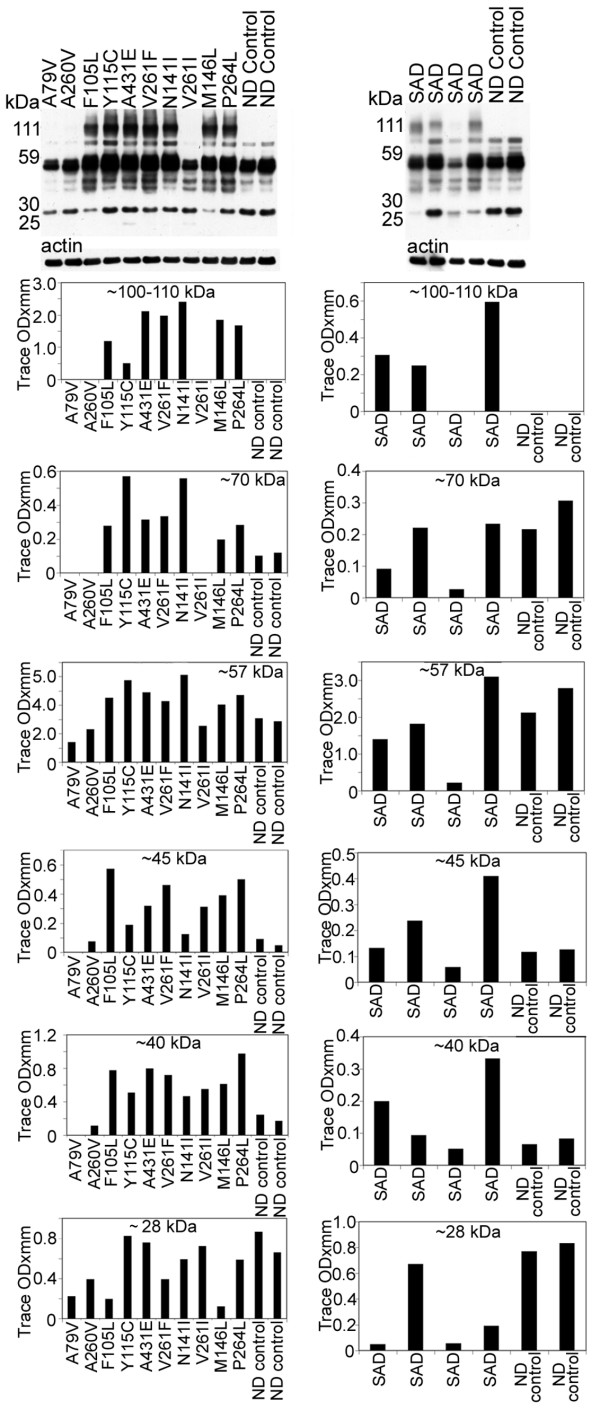
**Western blots of soluble tau isoforms quantified by scanning densitometry**. The overall quantities of soluble tau isoforms substantially varied among the different PSEN mutations and SAD cases and from the ND controls. Most of the tau proteins were concentrated between ~60–40 kDa. In general there was more tau protein in the PSEN mutations than in the ND controls. There were prominent bands at ~100–110 kDa in 7 out of 10 PS mutations that were absent in the ND controls. Likewise, this band was seen in 3 out of 4 of the SAD cases and not in the ND controls. These bands may correspond to dimeric forms of tau. SAD = sporadic Alzheimer's disease; ND = non-demented.

Tau antibody probing revealed a series of bands with a range of ~110 to ~28 kDa (Figure 4), representing both normal and PHF-tau [62]. In seven out of ten PSEN mutations there were bands that corresponded to dimeric forms of tau (~100–110 kDa). In general, the PSEN mutations demonstrated elevated amounts of soluble tau protein relative to the ND controls, the exception being the ~28 and ~57 kDa bands (Figure 4). The PSEN A79V and V261I mutations revealed low NFT counts on neuropathological examination (Table 2), and on WB had relatively moderate amounts of soluble tau peptides and were missing the ~100–110 kDa (dimeric) peptides (Figure 4). The case with the A260V mutation, which also was missing the ~100–110 kDa peptides, had an average amount NFT (31.00 per mm^2^) (Table 2). The PSEN cases with the highest NFT scores (A431E, V261F, and N141I; Table 2) also had the highest levels of dimeric tau as observed by WB (Figure 4).

### II. Characterization of AβPP, Notch, N-cadherin, Erb-B4 Processing

**Figure 5:**
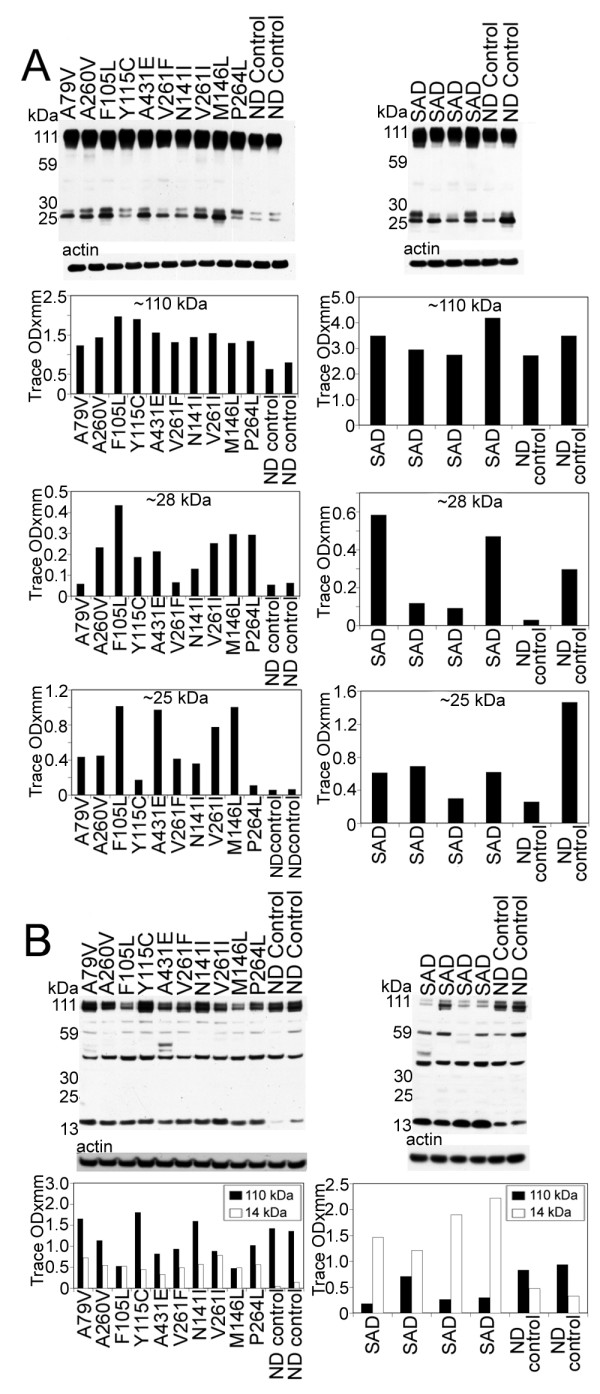
**Western blots of AβPP and N- and C-terminal related peptides**. A) The amounts of AβPP holoprotein (110 kDa) and 28 and 25 kDa bands are increased in all PSEN mutations relative to the ND controls. The 110 kDa (AβPP) in SAD is almost equivalent to the ND cases, but is variable with respect to the 28 and 25 kDa bands. B) In our SDS-PAGE system the CT99 and CT83 co-migrate as a single band at ~14 kDa. As can be appreciated, the ND controls have significantly less CT peptides than the PSEN and SAD cases. In addition, AβPP (110 kDa) was variable between the PSEN cases and the ND controls, but was decreased in the SAD cases compared to the ND controls. SAD = sporadic Alzheimer's disease; ND = non-demented.

The N- and C-terminal peptide degradation of AβPP was investigated by WB (Figure 5). The total amount of AβPP (~110 kDa), detected by the 22C11 antibody appears to be approximately increased two-fold among the PSEN mutations compared to ND controls. The total AβPP in SAD cases was the same or slightly elevated than those observed for ND controls (Figure 5A). The 28 and 25 kDa peptide bands were faint in ND cases and significantly elevated in the PSEN mutations in general, suggesting a larger accumulation of N-terminal peptides. A variable amount of N-terminal peptides were observed in SAD cases in relation to those of ND controls. The C-terminal (CT) peptides detected by the CT9APP antibody demonstrated a greater accumulation of the CT99/CT83 bands in PSEN mutations and SAD cases than those observed in ND controls. In contrast, SAD cases showed elevated quantities of CT peptides than those observed in both the ND and PSEN cohorts (Figure 5B). The levels of AβPP were increased compared to CT peptide levels in ND controls and PSEN cases. This ratio was inversed in the SAD group (Figure 5B). Intriguingly, there was a ~40 kDa band that our laboratory has routinely observed in other FAD, SAD, ND controls and transgenic mice which we are presently investigating.

**Figure 6:**
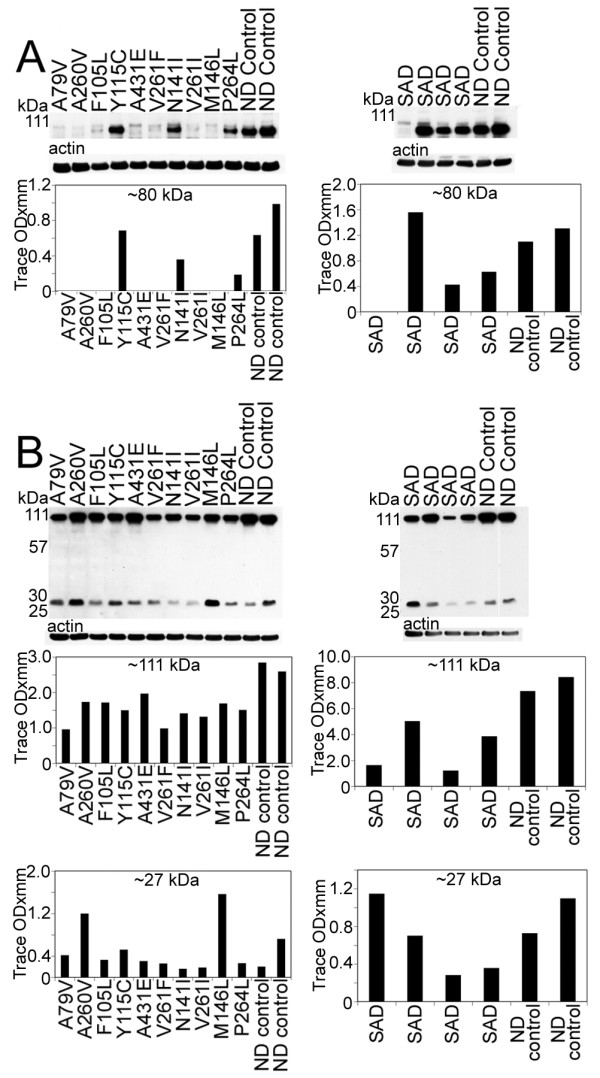
**Western blots of the 80 kDa Notch-1 intracellular domain (NICD) and N-cadherin/CTF2**.A) Notice that, with the exception of 3 PSEN mutation cases (Y115C/PSEN1, N141I/PSEN2 and P264L/PSEN1), all the PSEN mutations have reduced quantities of NICD transcription factor. In the SAD cases, there is a heterogeneous distribution of NICD, being almost negligible in one SAD case, elevated in a second case and diminished in the remaining two, relative to the ND controls. B) The amount of N-Cadherin holoprotein detected at 110 kDa is reduced in all PSEN cases relative to the ND controls. A similar pattern is observed for the SAD cases. The N-Cad/CTF2 band at 28 kDa is decreased in all PSEN mutations with the exception of A260V and M146L mutations that displayed higher values. The quantities of the N-Cad/CTF2 were variable in the SAD relative to the ND levels. SAD = sporadic Alzheimer's disease; ND = non-demented.

Analysis of Notch-1 by WB suggested that the S3 cleavage was negligible in seven of the ten PSEN mutations, since the levels of the ~80 kDa NICD were very low. However, we cannot exclude the possibility of a faster turnover of the fragment resulting in lower steady-state levels. Two of the remaining three cases (P264L and N141I), exhibited lesser amounts of the 80 kDa NICD when compared to ND controls, while the remaining case (Y115C) had comparable values to ND controls (Figure 6A). The detected amounts of the NICD peptide in SAD were very heterogeneous. In one case only trace amounts of NICD were present. In two cases this peptide existed at lower levels than those in ND controls while in one individual the level was higher than the ND controls (Figure 6A).

Our WB data demonstrated both full-length N-cadherin (110 kDa) and the CT fragment (28 kDa), as observed in other laboratories [46,63,64]. The level of full-length N-cadherin was decreased in all PSEN mutation carriers as well as in SAD cases when compared to ND controls (Figure 6B). With the exception of two PSEN1 mutations (A260V and M146L), the remaining PSEN mutations and two ND controls showed variable amounts of the ~28 kDa peptide. This pattern was also observed in the SAD cohort (Figure 6B).

**Figure 7:**
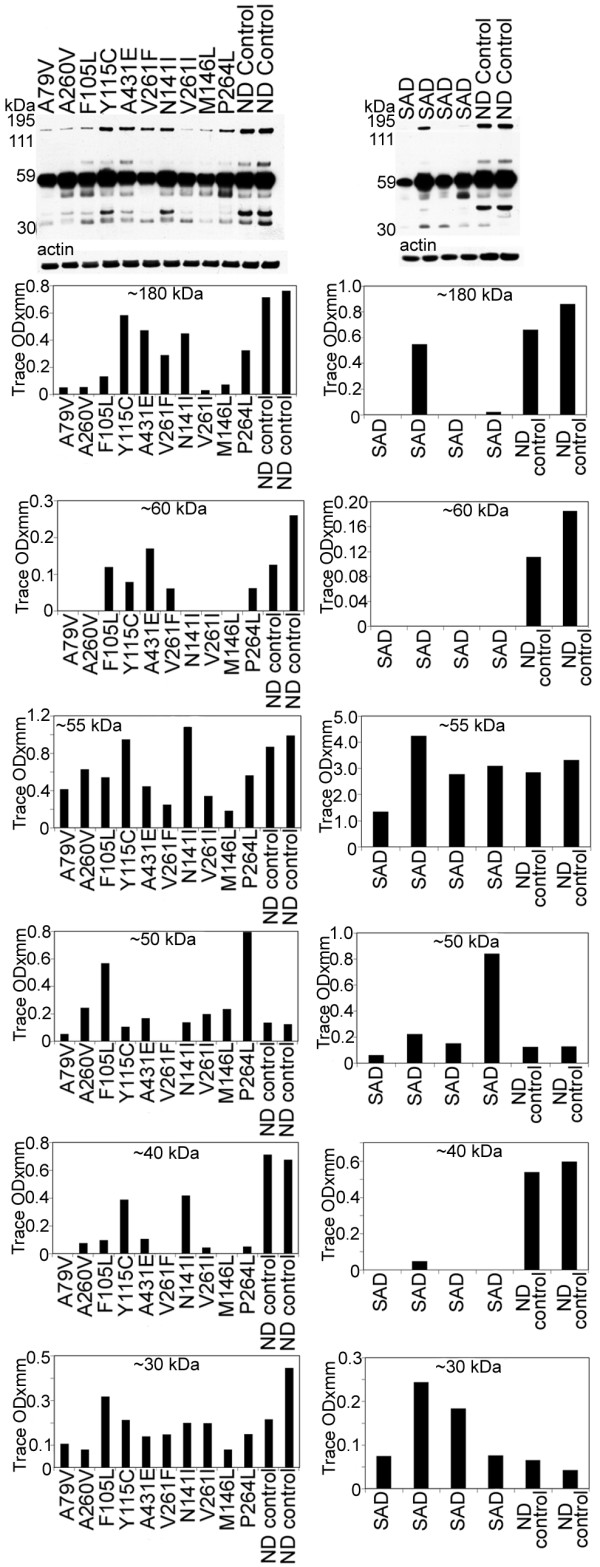
**Western blots of Erb-B4**. Overall, the bands corresponding to the holoprotein (~180 kDa) and ~60 kDa were decreased in all PSEN mutations as well as in the SAD cases relative to the ND controls. Of the degradation peptides that carry the Erb-B4 C-terminal epitope the most represented band, at ~55 kDa, demonstrated heterogeneous levels among different PSEN mutations. In the SAD cases the values were more similar to the control levels. The ~50 and ~30 kDa peptide bands also show large deviations among the PSEN, SAD and ND controls. The ~40 kDa band was increased in the ND controls when compared to the PSEN and SAD cases. SAD = sporadic Alzheimer's disease; ND = non-demented.

The total levels of Erb-B4 (~180 kDa) were reduced in the PSEN mutations, relative to ND cases as revealed by WB. A similar pattern was observed in the SAD cohort (Figure 7). In PSEN and SAD individuals there was a reduction of the ~60 kDa peptide. The most prominent band (~55 kDa) showed variable quantities among the PSEN mutations and ND controls. Cases F105L and P264L had the highest amounts of the ~50 kDa band with the remaining PSEN cases and ND controls having similar quantities. In most of the PSEN mutations the ~40 kDa band was 2–10 fold diminished, while the ~30 kDa peptide had heterogeneous values (Figure 7). Absorption experiments by ourselves (data not shown) and others [65,66], revealed that the fragments were specific to Erb-B4. Overall, the WB patterns suggest that in the PSEN mutations, Erb-B4 has a high degree of variation in CT peptide degradation (Figure 7).

## Discussion

One of the most significant characteristics of the ten studied PSEN mutations was their early age of clinical onset (mean: 44.7 years, range 35–60), which confirms previous observations (reviewed in reference [67]). Younger SAD individuals (mean: 64 years, range 55–73) were used in the study in order to make the comparisons more equivalent. The different PSEN mutations exhibited substantial heterogeneity in duration of the disease, degree of brain atrophy, neuronal loss, cortical and leptomeningeal vascular amyloidosis and basic type, number and distribution of amyloid plaques. Likewise, the NFT demonstrated a wide range of variation in amount and distribution among the ten PSEN mutation individuals. These sharply different pathological manifestations suggest that a single genetic change in the *PSEN *genes results in the phenotypic variations associated with each distinct PSEN mutations due to complex pleiotropic effects.

It is widely accepted that a major pathological effect of PSEN mutations is a proportionately larger production of toxic Aβ42 [15-22]. In our study, this hypothesis was supported by the high Aβ42/Aβ40 ratio observed in six PSEN mutation cases. However, in four instances PSEN mutations resulted in Aβ40 levels that were substantially higher than those of Aβ42. In terms of mean ng/g of cerebral cortex, the PSEN mutations had significantly more Aβ40 (7,093) than Aβ42 (4,856), while the SAD cases had less Aβ40 (2,627) than Aβ42 (3,322) and the ND cases had very small amounts of both Aβ40 (42) and Aβ42 (132). These differences may, in part, be due to a larger amount of cerebrovascular amyloidosis in which Aβ40 deposits predominate. However, previous PSEN studies in which the ratio of Aβ42/Aβ40 was elevated were mostly performed in either cell cultures or in mice expressing mutated *PSEN *genes [23]. Vascular amyloidosis plays a fundamental pathological role in PSEN dementias and in FAD and SAD by altering blood-brain barrier permeability and obstructing the small cerebral vessels. In larger arteries it obliterates the tunica media with loss of compliance, thus decreasing brain perfusion. Furthermore, severe perivascular amyloid deposition blocks the periarterial spaces that remove the interstitial fluid of the brain into the systemic circulation, resulting in dilation of the periarterial spaces, brain edema and grave hemodynamic alterations [68,69]. Isolated leptomeninges from PSEN mutation cases also displayed a wide variation in amyloid angiopathy.

We observed no elevation in the frequency of *APOE *ε4 allele as it occurs in SAD, suggesting that this allele may not play a pivotal role in the PSEN phenotype. However, a larger population of PSEN mutations will need to be studied to endorse such a conclusion. Interestingly, in the PSEN1 mutation group, those two individuals that carried the *APOE *ε2/ε3 genotype (A260V and F105L mutations) had the lowest amount of total Aβ in the frontal cortex, averaging 1,533 ng/g of tissue. By contrast, the remaining PSEN mutation subjects had an average total Aβ of 14,554 ng/g of tissue, a 9-fold greater level. These sharp differences suggest that the presence of *APOE *ε2 allele may play a role in limiting Aβ production and/or its accumulation.

Total levels of soluble tau protein were increased in many PSEN mutation cases compared to ND controls. Interestingly, seven out of the ten PSEN mutations exhibited dimeric tau levels as did three out of four SAD cases. None of the control cases showed dimeric tau. Additionally, two out of three PSEN cases that did not have the dimeric tau bands, had the two lowest NFT neuropathology counts. The remaining case had an average amount of NFT. The cases that had the three highest NFT counts also had the highest dimeric tau values as observed by WB. These findings can be explained by the fact that dimerization of tau is pivotal in nucleation and in paired filament elongation [70]. Given that NFT are an important hallmark of AD, since they obliterate neurons and their neuritic arbors and are in part responsible for the dementing process in PSEN mutations, it is surprising to find a wide degree of heterogeneity in soluble tau levels and insoluble NFT pathology. Based on previous studies, abnormal phosphorylation of soluble tau apparently results in filament formation, suggesting that phosphorylation may be a driving force in NFT assembly [71,72].

There was an interesting contrast between the amount of AβPP and CT peptides in the PSEN mutations as the opposite situation was shown for the SAD cases, where the amount of CT peptides was by far elevated relative to the AβPP. The reversed ratios imply an accumulation of AβPP in most of the PSEN mutations, probably due to defective γ-secretase that limits the generation of Aβ and AICD. Paradoxically, in SAD cases, the presence of abundant CT peptides, suggests a very active β-secretase with an accumulation of CT fragments relative to ND control levels. The elevation of CT peptides in PSEN and SAD cases compared to ND controls is also of interest because one would expect these fragments to be diminished, or at least to be found at the levels of the ND cases, in view of the universal accumulation of Aβ in the SAD individuals. An increase in CT fragments may be a toxic factor in PSEN dementias and SAD as happens in animal models. In transgenic mice expressing the CT100 amino acid domain of AβPP, there are neuronal and synaptic pathologies, intracellular accumulation of CT fragments, hippocampal degeneration, cerebrovascular alterations and cognitive deficits [73-76]. The accumulation of Aβ in PSEN and SAD individuals is probably due to decreased enzymatic degradation and decreased clearance of these molecules into the systemic circulation. It has recently been suggested that a single *PSEN*-affected gene may act as a dominant-negative mutant capable of inhibiting the normal activity of other molecules, such as cAMP-response element (CRE)-dependent gene expression, as well as NMDA receptor function and synaptic plasticity leading to cell death [77]. It is also possible that structural changes resulting from PSEN mutations may generate longer than Aβ42 peptides that remain membrane bound and have a toxic cumulative effect [78]. In addition, PSEN mutations may increase the accumulation of AβCT terminal peptides [79]. There are two possible mechanisms for the observed accumulation of AβPP in the PSEN mutations. First, the increased levels may be due to a failure of the mutated PSEN to cleave AβPP and yield Aβ peptides. Second, it has been suggested that PSEN1 is also a regulator of β-secretase [80] and therefore its dysfunction could result in the accumulation of AβPP.

Mutations in the *PSEN *genes result in a complicated pleiotropic cascade since they generate a large number of aberrantly processed molecules and factors that profoundly alter brain function and manifest in an early disease onset, a wide range of clinical symptoms and diverse final neuropathology. Important in this pathology are the peptides derived from γ-secretase hydrolysis that are essential in structural and regulatory functions such as those produced by AβPP, Notch-1, N-Cadherin and Erb-B4. Notch processing appeared to be impaired in all ten PSEN cases compared to ND cases, but in seven cases this effect was profound with an almost complete absence of detectable NICD. A direct role for PSEN mutations in the apparent reduction of NICD expression has yet to be established. However, given the large number of genes that are activated by the NICD/CSL transcription factor, important pathophysiological changes occur that may play a large role in the development of these dementias. In contrast to ND cases, all PSEN mutations and SAD individuals demonstrated a reduced amount of N-Cadherin, which in part may be due to either mutations in *PSEN *genes or to limited function of γ-secretase. Aberrant processing of N-Cadherin would trigger the accumulation of CBP resulting in damage to CREB transcriptional function and in impaired synaptic transmission and synaptic morphogenesis and ensuing cognitive decline [81,82]. Erb-B4 and B4ICD domain levels were lower in all PSEN and SAD cases in comparison to ND controls. Synthesis of both peptides may be limited by mutations in γ-secretase, as it occurs with the inhibition of γ-secretase in tissue culture cells that results in the accumulation of CT fragments [52]. Defective processing of Erb-B4 by γ-secretase may cause deviant endoproteolysis, thus explaining the multiple Erb-B4 CT related peptides observed in our WB. It has been suggested that a defective balance between AβPP an Erb-B4 hydrolysis may contribute to neurodegeneration, memory deficits and synaptic dysfunction without generating amyloid plaques [83]. In addition, a decrease in Erb-B4 leads to the up-regulation of GFAP that may enhance the astrocytosis observed in SAD [84].

## Conclusion

The early onset and morphological and biochemical diversity observed among the PSEN mutations are obviously related to the particular site and type of mutation. The fact that SAD and PSEN mutations both accumulate Aβ, suggest that AβPP and Aβ peptides play an important role in brain aging. Furthermore, AβPP mutations in the inherited forms of FAD that flank or lie within the Aβ sequence, underscore the potential metabolic importance of the CT99 and Aβ peptides. This suggests that Aβ may represent a rescue molecule in different neurodegenerative pathologies [85]. Therapeutic interventions, such as Aβ immunization as a prophylactic AD venue, should be administered to younger clinically healthy carriers of PSEN mutation families, to assess the effects of preventing Aβ deposition and to clinically evaluate to which extent Aβ is responsible for the dementia observed in AD.

Presenilin mutations, although inducing an early-onset dementia with overall similarity to SAD, exhibit a range of pathologic and clinical phenotypes. This complexity belies the simple explanatory hypothesis that suggests a straightforward preferential production of Aβ42 as a sole underlying biochemical mechanism. Systematic examination of 10 PSEN mutation cases revealed that only half exhibited substantially increased Aβ42:Aβ40 ratios. This suggests that neither increased production nor enhanced accumulation of Aβ42 accounts for the range of pathological effects resulting from a large number of altered PSEN functions. In view of these differences, the multiple substrates affected, early age of onset and clinical course and the presentation of a different therapeutic target, FAD due to PSEN mutations may be better classified as "Presenilin Dementias," with the caveat that more information is needed to justify their departure from FAD.

## Abbreviations

AβPP: amyloid-beta precursor protein; Aβ: amyloid beta; AD: Alzheimer's disease; ADAM: A disintegrin and metalloproteinase; AICD: amyloid-beta intracellular domain; Aph1: anterior pharynx defective 1; Asp: aspartic acid; APOE: apolipoprotein E; B4ICD: B4 intracellular domain; CAA: cerebral amyloid angiopathy; CREB: cAMP response element binding protein; CBP: CREB binding protein; CSL: CBF1/Suppressor of Hairless/Lag1; CT: C-terminal; CWP: cotton wool plaques; EGF: epidermal growth factor; ELISA: enzyme-linked immunoabsorbent assay; FAD: familial Alzheimer's disease; GDFA: glass distilled formic acid; GFAP: glial fibrillary acidic protein; N-Cad/CTF2: N-cadherin/C-terminal fragment 2; ND: non-demented; NFT: neurofibrillary tangles; NICD: Notch intracellular domain; NRG: neuroregulins; MES: morpholinoethanesulfonic acid; PBS: phosphate-buffered saline; PCR: polymerase chain reaction; Pen2: presenilin enhancer 2; PHF: paired helical filaments; PMI: postmortem interval; PSEN: presenilin; SAD: sporadic Alzheimer's disease; TACE: tumor necrosis-α-converting enzyme; TMD: transmembrane domain; WB: Western blot.

## Competing interests

The authors declare that they have no competing interests.

## Authors' contributions

CLM participated in the design, experimental work and production of manuscript. IDD carried out the experimental work. SS and RV performed the neuropathology assessment. TAK was involved in the design and production of manuscript. RLP, WMK and DCL participated in the experimental work and editing. DGW performed the Apo E genotyping. EMC was involved in the design and manuscript production. TGB and BG participated in the neuropathology assessment and manuscript production. AER was responsible for the overall design and production of the manuscript. All authors read and approved the final manuscript.

## References

[B1] Kopan R, Goate A (2000). A common enzyme connects notch signaling and Alzheimer's disease. Genes Dev.

[B2] Spasic D, Tolia A, Dillen K, Baert V, De Strooper B, Vrijens S, Annaert W (2006). Presenilin-1 maintains a nine-transmembrane topology throughout the secretory pathway. J Biol Chem.

[B3] Wolfe MS, Xia W, Ostaszewski BL, Diehl TS, Kimberly WT, Selkoe DJ (1999). Two transmembrane aspartates in presenilin-1 required for presenilin endoproteolysis and gamma-secretase activity. Nature.

[B4] Kimberly WT, LaVoie MJ, Ostaszewski BL, Ye W, Wolfe MS, Selkoe DJ (2003). Gamma-secretase is a membrane protein complex comprised of presenilin, nicastrin, Aph-1, and Pen-2. Proc Natl Acad Sci USA.

[B5] Wolfe MS (2006). The gamma-secretase complex: membrane-embedded proteolytic ensemble. Biochemistry.

[B6] Hebert SS, Serneels L, Dejaegere T, Horre K, Dabrowski M, Baert V, Annaert W, Hartmann D, De Strooper B (2004). Coordinated and widespread expression of gamma-secretase in vivo: evidence for size and molecular heterogeneity. Neurobiol Dis.

[B7] Selkoe DJ, Wolfe MS (2007). Presenilin: running with scissors in the membrane. Cell.

[B8] Parks AL, Curtis D (2007). Presenilin diversifies its portfolio. Trends Genet.

[B9] Handler M, Yang X, Shen J (2000). Presenilin-1 regulates neuronal differentiation during neurogenesis. Development.

[B10] Nishimura M, Yu G, Levesque G, Zhang DM, Ruel L, Chen F, Milman P, Holmes E, Liang Y, Kawarai T, Jo E, Supala A, Rogaeva E, Xu DM, Janus C, Levesque L, Bi Q, Duthie M, Rozmahel R, Mattila K, Lannfelt L, Westaway D, Mount HT, Woodgett J, Fraser PE, St George-Hyslop P (1999). Presenilin mutations associated with Alzheimer disease cause defective intracellular trafficking of beta-catenin, a component of the presenilin protein complex. Nat Med.

[B11] Zhang Z, Hartmann H, Do VM, Abramowski D, Sturchler-Pierrat C, Staufenbiel M, Sommer B, Wetering M van de, Clevers H, Saftig P, De Strooper B, He X, Yankner BA (1998). Destabilization of beta-catenin by mutations in presenilin-1 potentiates neuronal apoptosis. Nature.

[B12] Weidemann A, Eggert S, Reinhard FB, Vogel M, Paliga K, Baier G, Masters CL, Beyreuther K, Evin G (2002). A novel epsilon-cleavage within the transmembrane domain of the Alzheimer amyloid precursor protein demonstrates homology with Notch processing. Biochemistry.

[B13] Davis JA, Naruse S, Chen H, Eckman C, Younkin S, Price DL, Borchelt DR, Sisodia SS, Wong PC (1998). An Alzheimer's disease-linked PS1 variant rescues the developmental abnormalities of PS1-deficient embryos. Neuron.

[B14] Kumar-Singh S, Theuns J, Van Broeck B, Pirici D, Vennekens K, Corsmit E, Cruts M, Dermaut B, Wang R, Van Broeckhoven C (2006). Mean age-of-onset of familial Alzheimer disease caused by presenilin mutations correlates with both increased Abeta42 and decreased Abeta40. Hum Mutat.

[B15] Borchelt DR, Thinakaran G, Eckman CB, Lee MK, Davenport F, Ratovitsky T, Prada CM, Kim G, Seekins S, Yager D, Slunt HH, Wang R, Seeger M, Levey AI, Gandy SE, Copeland NG, Jenkins NA, Price DL, Younkin SG, Sisodia SS (1996). Familial Alzheimer's disease-linked presenilin 1 variants elevate Abeta1-42/1-40 ratio in vitro and in vivo. Neuron.

[B16] Duff K, Eckman C, Zehr C, Yu X, Prada CM, Perez-tur J, Hutton M, Buee L, Harigaya Y, Yager D, Morgan D, Gordon MN, Holcomb L, Refolo L, Zenk B, Hardy J, Younkin S (1996). Increased amyloid-beta42(43) in brains of mice expressing mutant presenilin 1. Nature.

[B17] Scheuner D, Eckman C, Jensen M, Song X, Citron M, Suzuki N, Bird TD, Hardy J, Hutton M, Kukull W, Larson E, Levy-Lahad E, Viitanen M, Peskind E, Poorkaj P, Schellenberg G, Tanzi R, Wasco W, Lannfelt L, Selkoe D, Younkin S (1996). Secreted amyloid beta-protein similar to that in the senile plaques of Alzheimer's disease is increased in vivo by the presenilin 1 and 2 and APP mutations linked to familial Alzheimer's disease. Nat Med.

[B18] Citron M, Westaway D, Xia W, Carlson G, Diehl T, Levesque G, Johnson-Wood K, Lee M, Seubert P, Davis A, Kholodenko D, Motter R, Sherrington R, Perry B, Yao H, Strome R, Lieberburg I, Rommens J, Kim S, Schenk D, Fraser P, St George HP, Selkoe DJ (1997). Mutant presenilins of Alzheimer's disease increase production of 42-residue amyloid beta-protein in both transfected cells and transgenic mice. Nat Med.

[B19] Xia W, Zhang J, Kholodenko D, Citron M, Podlisny MB, Teplow DB, Haass C, Seubert P, Koo EH, Selkoe DJ (1997). Enhanced production and oligomerization of the 42-residue amyloid beta-protein by Chinese hamster ovary cells stably expressing mutant presenilins. J Biol Chem.

[B20] Murayama O, Tomita T, Nihonmatsu N, Murayama M, Sun X, Honda T, Iwatsubo T, Takashima A (1999). Enhancement of amyloid beta 42 secretion by 28 different presenilin 1 mutations of familial Alzheimer's disease. Neurosci Lett.

[B21] Houlden H, Baker M, McGowan E, Lewis P, Hutton M, Crook R, Wood NW, Kumar-Singh S, Geddes J, Swash M, Scaravilli F, Holton JL, Lashley T, Tomita T, Hashimoto T, Verkkoniemi A, Kalimo H, Somer M, Paetau A, Martin JJ, Van Broeckhoven C, Golde T, Hardy J, Haltia M, Revesz T (2000). Variant Alzheimer's disease with spastic paraparesis and cotton wool plaques is caused by PS-1 mutations that lead to exceptionally high amyloid-beta concentrations. Ann Neurol.

[B22] Jankowsky JL, Fadale DJ, Anderson J, Xu GM, Gonzales V, Jenkins NA, Copeland NG, Lee MK, Younkin LH, Wagner SL, Younkin SG, Borchelt DR (2004). Mutant presenilins specifically elevate the levels of the 42 residue beta-amyloid peptide in vivo: evidence for augmentation of a 42-specific gamma secretase. Hum Mol Genet.

[B23] Wolfe MS (2007). When loss is gain: reduced presenilin proteolytic function leads to increased Abeta42/Abeta40. Talking point on the role of presenilin mutations in Alzheimer disease. EMBO Rep.

[B24] Shioi J, Georgakopoulos A, Mehta P, Kouchi Z, Litterst CM, Baki L, Robakis NK (2007). FAD mutants unable to increase neurotoxic Abeta 42 suggest that mutation effects on neurodegeneration may be independent of effects on Abeta. J Neurochem.

[B25] De Strooper B (2007). Loss-of-function presenilin mutations in Alzheimer disease. Talking Point on the role of presenilin mutations in Alzheimer disease. EMBO Rep.

[B26] Chen Q, Nakajima A, Choi SH, Xiong X, Tang YP (2008). Loss of presenilin function causes Alzheimer's disease-like neurodegeneration in the mouse. J Neurosci Res.

[B27] Goux WJ, Rodriguez S, Sparkman DR (1995). Analysis of the core components of Alzheimer paired helical filaments. A gas chromatography/mass spectrometry characterization of fatty acids, carbohydrates and long-chain bases. FEBS Lett.

[B28] Goux WJ, Rodriguez S, Sparkman DR (1996). Characterization of the glycolipid associated with Alzheimer paired helical filaments. J Neurochem.

[B29] Goux WJ, Liu B, Shumburo AM, Parikh S, Sparkman DR (2001). A quantitative assessment of glycolipid and protein associated with paired helical filament preparations from Alzheimer's diseased brain. J Alzheimers Dis.

[B30] Clements JR, Beitz AJ, Emory CR, Frey WH (1990). Immunogold labeling of Alzheimer paired helical filaments with ganglioside MAB A2B5. Alzheimer Dis Assoc Disord.

[B31] Gray EG, Paula-Barbosa M, Roher A (1987). Alzheimer's disease: paired helical filaments and cytomembranes. Neuropathol Appl Neurobiol.

[B32] Farah CA, Perreault S, Liazoghli D, Desjardins M, Anton A, Lauzon M, Paiement J, Leclerc N (2006). Tau interacts with Golgi membranes and mediates their association with microtubules. Cell Motil Cytoskeleton.

[B33] Fiuza UM, Arias AM (2007). Cell and molecular biology of Notch. J Endocrinol.

[B34] Bray SJ (2006). Notch signalling: a simple pathway becomes complex. Nat Rev Mol Cell Biol.

[B35] Lai EC (2004). Notch signaling: control of cell communication and cell fate. Development.

[B36] Shih I, Wang TL (2007). Notch signaling, gamma-secretase inhibitors, and cancer therapy. Cancer Res.

[B37] Bertagna A, Toptygin D, Brand L, Barrick D (2008). The effects of conformational heterogeneity on the binding of the Notch intracellular domain to effector proteins: a case of biologically tuned disorder. Biochem Soc Trans.

[B38] Tagami S, Okochi M, Yanagida K, Ikuta A, Fukumori A, Matsumoto N, Ishizuka-Katsura Y, Nakayama T, Itoh N, Jiang J, Nishitomi K, Kamino K, Morihara T, Hashimoto R, Tanaka T, Kudo T, Chiba S, Takeda M (2008). Regulation of Notch signaling by dynamic changes in the precision of S3 cleavage of Notch-1. Mol Cell Biol.

[B39] Iso T, Kedes L, Hamamori Y (2003). HES and HERP families: multiple effectors of the Notch signaling pathway. J Cell Physiol.

[B40] Martinez Arias A, Zecchini V, Brennan K (2002). CSL-independent Notch signalling: a checkpoint in cell fate decisions during development?. Curr Opin Genet Dev.

[B41] Yap AS, Brieher WM, Pruschy M, Gumbiner BM (1997). Lateral clustering of the adhesive ectodomain: a fundamental determinant of cadherin function. Curr Biol.

[B42] Yap AS, Brieher WM, Gumbiner BM (1997). Molecular and functional analysis of cadherin-based adherens junctions. Annu Rev Cell Dev Biol.

[B43] Derycke LD, Bracke ME (2004). N-cadherin in the spotlight of cell-cell adhesion, differentiation, embryogenesis, invasion and signalling. Int J Dev Biol.

[B44] Bruses JL (2006). N-cadherin signaling in synapse formation and neuronal physiology. Mol Neurobiol.

[B45] Hirano S, Suzuki ST, Redies C (2003). The cadherin superfamily in neural development: diversity, function and interaction with other molecules. Front Biosci.

[B46] Marambaud P, Wen PH, Dutt A, Shioi J, Takashima A, Siman R, Robakis NK (2003). A CBP binding transcriptional repressor produced by the PS1/epsilon-cleavage of N-cadherin is inhibited by PS1 FAD mutations. Cell.

[B47] Francoeur JR, Richardson PM, Dunn RJ, Carbonetto S (1995). Distribution of erb-B2, erb-B3, and erb-B4 in the developing avian nervous system. J Neurosci Res.

[B48] Carpenter G (2003). ErbB-4: mechanism of action and biology. Exp Cell Res.

[B49] Vidal GA, Naresh A, Marrero L, Jones FE (2005). Presenilin-dependent gamma-secretase processing regulates multiple ERBB4/HER4 activities. J Biol Chem.

[B50] Ni CY, Yuan H, Carpenter G (2003). Role of the ErbB-4 carboxyl terminus in gamma-secretase cleavage. J Biol Chem.

[B51] Cheng QC, Tikhomirov O, Zhou W, Carpenter G (2003). Ectodomain cleavage of ErbB-4: characterization of the cleavage site and m80 fragment. J Biol Chem.

[B52] Lee HJ, Jung KM, Huang YZ, Bennett LB, Lee JS, Mei L, Kim TW (2002). Presenilin-dependent gamma-secretase-like intramembrane cleavage of ErbB4. J Biol Chem.

[B53] Zhang YW, Wang R, Liu Q, Zhang H, Liao FF, Xu H (2007). Presenilin/gamma-secretase-dependent processing of beta-amyloid precursor protein regulates EGF receptor expression. Proc Natl Acad Sci USA.

[B54] Chaudhury AR, Gerecke KM, Wyss JM, Morgan DG, Gordon MN, Carroll SL (2003). Neuregulin-1 and erbB4 immunoreactivity is associated with neuritic plaques in Alzheimer disease brain and in a transgenic model of Alzheimer disease. J Neuropathol Exp Neurol.

[B55] Falls DL (2003). Neuregulins: functions, forms, and signaling strategies. Exp Cell Res.

[B56] Murphy S, Krainock R, Tham M (2002). Neuregulin signaling via erbB receptor assemblies in the nervous system. Mol Neurobiol.

[B57] Beach TG, Sue LI, Walker DG, Roher AE, Lue L, Vedders L, Connor DJ, Sabbagh MN, Rogers J (2008). The Sun Health Research Institute Brain Donation Program: Description and Experience, 1987–2007. Cell Tissue Bank.

[B58] Armstrong RA (1998). Beta-amyloid plaques: stages in life history or independent origin?. Dement Geriatr Cogn Disord.

[B59] Miravalle L, Calero M, Takao M, Roher AE, Ghetti B, Vidal R (2005). Amino-terminally truncated Abeta peptide species are the main component of cotton wool plaques. Biochemistry.

[B60] Harrington CR, Louwagie J, Rossau R, Vanmechelen E, Perry RH, Perry EK, Xuereb JH, Roth M, Wischik CM (1994). Influence of apolipoprotein E genotype on senile dementia of the Alzheimer and Lewy body types. Significance for etiological theories of Alzheimer's disease. Am J Pathol.

[B61] Hixson JE, Vernier DT (1990). Restriction isotyping of human apolipoprotein E by gene amplification and cleavage with HhaI. J Lipid Res.

[B62] King ME, Gamblin TC, Kuret J, Binder LI (2000). Differential assembly of human tau isoforms in the presence of arachidonic acid. J Neurochem.

[B63] Uemura K, Kuzuya A, Aoyagi N, Ando K, Shimozono Y, Ninomiya H, Shimohama S, Kinoshita A (2007). Amyloid beta inhibits ectodomain shedding of N-cadherin via down-regulation of cell-surface NMDA receptor. Neuroscience.

[B64] Uemura K, Kihara T, Kuzuya A, Okawa K, Nishimoto T, Bito H, Ninomiya H, Sugimoto H, Kinoshita A, Shimohama S (2006). Activity-dependent regulation of beta-catenin via epsilon-cleavage of N-cadherin. Biochem Biophys Res Commun.

[B65] Thompson M, Lauderdale S, Webster MJ, Chong VZ, McClintock B, Saunders R, Weickert CS (2007). Widespread expression of ErbB2, ErbB3 and ErbB4 in non-human primate brain. Brain Res.

[B66] Chong VZ, Thompson M, Beltaifa S, Webster MJ, Law AJ, Weickert CS (2008). Elevated neuregulin-1 and ErbB4 protein in the prefrontal cortex of schizophrenic patients. Schizophr Res.

[B67] Larner AJ, Doran M (2006). Clinical phenotypic heterogeneity of Alzheimer's disease associated with mutations of the presenilin-1 gene. J Neurol.

[B68] Roher AE, Kuo YM, Esh C, Knebel C, Weiss N, Kalback W, Luehrs DC, Childress JL, Beach TG, Weller RO, Kokjohn TA (2003). Cortical and leptomeningeal cerebrovascular amyloid and white matter pathology in Alzheimer's disease. Mol Med.

[B69] Kalback W, Esh C, Castano EM, Rahman A, Kokjohn T, Luehrs DC, Sue L, Cisneros R, Gerber F, Richardson C, Bohrmann B, Walker DG, Beach TG, Roher AE (2004). Atherosclerosis, vascular amyloidosis and brain hypoperfusion in the pathogenesis of sporadic Alzheimer's disease. Neurol Res.

[B70] Friedhoff P, von Bergen M, Mandelkow EM, Davies P, Mandelkow E (1998). A nucleated assembly mechanism of Alzheimer paired helical filaments. Proc Natl Acad Sci USA.

[B71] Iqbal K, Grundke-Iqbal I (2006). Discoveries of tau, abnormally hyperphosphorylated tau and others of neurofibrillary degeneration: a personal historical perspective. J Alzheimers Dis.

[B72] Goedert M, Klug A, Crowther RA (2006). Tau protein, the paired helical filament and Alzheimer's disease. J Alzheimers Dis.

[B73] Berger-Sweeney J, McPhie DL, Arters JA, Greenan J, Oster-Granite ML, Neve RL (1999). Impairments in learning and memory accompanied by neurodegeneration in mice transgenic for the carboxyl-terminus of the amyloid precursor protein. Brain Res Mol Brain Res.

[B74] McPhie DL, Lee RK, Eckman CB, Olstein DH, Durham SP, Yager D, Younkin SG, Wurtman RJ, Neve RL (1997). Neuronal expression of beta-amyloid precursor protein Alzheimer mutations causes intracellular accumulation of a C-terminal fragment containing both the amyloid beta and cytoplasmic domains. J Biol Chem.

[B75] Oster-Granite ML, McPhie DL, Greenan J, Neve RL (1996). Age-dependent neuronal and synaptic degeneration in mice transgenic for the C terminus of the amyloid precursor protein. J Neurosci.

[B76] Neve RL, Boyce FM, McPhie DL, Greenan J, Oster-Granite ML (1996). Transgenic mice expressing APP-C100 in the brain. Neurobiol Aging.

[B77] Shen J, Kelleher RJ (2007). The presenilin hypothesis of Alzheimer's disease: evidence for a loss-of-function pathogenic mechanism. Proc Natl Acad Sci USA.

[B78] Van Vickle GD, Esh CL, Kokjohn TA, Patton RL, Kalback WM, Luehrs DC, Beach TG, Newel AJ, Lopera F, Ghetti B, Vidal R, Castano EM, Roher AE (2008). Presenilin-1 280Glu-->Ala mutation alters C-terminal APP processing yielding longer Abeta peptides: Implications for Alzheimer's disease. Mol Med.

[B79] Sato T, Dohmae N, Qi Y, Kakuda N, Misonou H, Mitsumori R, Maruyama H, Koo EH, Haass C, Takio K, Morishima-Kawashima M, Ishiura S, Ihara Y (2003). Potential link between amyloid beta-protein 42 and C-terminal fragment gamma 49–99 of beta-amyloid precursor protein. J Biol Chem.

[B80] Kuzuya A, Uemura K, Kitagawa N, Aoyagi N, Kihara T, Ninomiya H, Ishiura S, Takahashi R, Shimohama S (2007). Presenilin 1 is involved in the maturation of beta-site amyloid precursor protein-cleaving enzyme 1 (BACE1). J Neurosci Res.

[B81] Marambaud P, Robakis NK (2005). Genetic and molecular aspects of Alzheimer's disease shed light on new mechanisms of transcriptional regulation. Genes Brain Behav.

[B82] Uemura K, Kuzuya A, Shimohama S (2004). Protein trafficking and Alzheimer's disease. Curr Alzheimer Res.

[B83] Saura CA, Choi SY, Beglopoulos V, Malkani S, Zhang D, Shankaranarayana Rao BS, Chattarji S, Kelleher RJ, Kandel ER, Duff K, Kirkwood A, Shen J (2004). Loss of presenilin function causes impairments of memory and synaptic plasticity followed by age-dependent neurodegeneration. Neuron.

[B84] Sardi SP, Murtie J, Koirala S, Patten BA, Corfas G (2006). Presenilin-dependent ErbB4 nuclear signaling regulates the timing of astrogenesis in the developing brain. Cell.

[B85] Heininger K (2000). A unifying hypothesis of Alzheimer's disease. IV. Causation and sequence of events. Rev Neurosci.

